# The temporal window of ecological adaptation in postglacial lakes: a comparison of head morphology, trophic position and habitat use in Norwegian threespine stickleback populations

**DOI:** 10.1186/s12862-016-0676-2

**Published:** 2016-05-13

**Authors:** Kjartan Østbye, Chris Harrod, Finn Gregersen, Tom Klepaker, Michael Schulz, Dolph Schluter, Leif Asbjørn Vøllestad

**Affiliations:** Department of Biosciences, Centre for Ecological and Evolutionary Synthesis (CEES), University of Oslo, P. O. Box 1066, Blindern, N-0316 Oslo Norway; Faculty of Applied Ecology and Agricultural Sciences, Hedmark University of Applied Science, Campus Evenstad, NO-2480 Koppang, Norway; Department of Physiological Ecology, Max Planck Institute for Limnology, Postfach 165, D-24302 Plön, Germany; Fish and Stable Isotope Ecology Laboratory, Instituto de Ciencias Naturales Alexander von Humbolt, Universidad de Antofagasta, Avenida Angamos 601, Antofagasta, Chile; Multiconsult, P.O. Box 265 Skøyen, N-0213 Oslo, Norway; Department of Biology, Aquatic Behavioural Ecology Research group, University of Bergen, P. O. Box 7800, N-5020 Bergen, Norway; August-Bebel-Strasse 46, 16816 Neuruppin, Germany; Department of Zoology, University of British Columbia, Vancouver, British Columbia V6T 1Z4 Canada

**Keywords:** Adaptive radiation, Natural selection, Ecological niche, Stable isotopic analysis, Isostatic uplift, Holocene, Pleistocene ice sheet, Benthic-limnetic stickleback species pairs

## Abstract

**Background:**

Studying how trophic traits and niche use are related in natural populations is important in order to understand adaptation and specialization. Here, we describe trophic trait diversity in twenty-five Norwegian freshwater threespine stickleback populations and their putative marine ancestor, and relate trait differences to postglacial lake age. By studying lakes of different ages, depths and distance to the sea we examine key environmental variables that may predict adaptation in trophic position and habitat use. We measured trophic traits including geometric landmarks that integrated variation in head shape as well as gillraker length and number. Trophic position (Tpos) and niche use (α) were estimated from stable isotopes (δ^13^C, δ^15^N). A comparison of head shape was also made with two North American benthic-limnetic species pairs.

**Results:**

We found that head shape differed between marine and freshwater sticklebacks, with marine sticklebacks having more upturned mouths, smaller eyes, larger opercula and deeper heads. Size-adjusted gillraker lengths were larger in marine than in freshwater stickleback. Norwegian sticklebacks were compared on the same head shape axis as the one differentiating the benthic-limnetic North American threespine stickleback species pairs. Here, Norwegian freshwater sticklebacks with a more “limnetic head shape” had more and longer gillrakers than sticklebacks with “benthic head shape”. The “limnetic morph” was positively associated with deeper lakes. Populations differed in α (mean ± sd: 0.76 ± 0.29) and Tpos (3.47 ± 0.27), where α increased with gillraker length. Larger fish had a higher Tpos than smaller fish. Compared to the ecologically divergent stickleback species pairs and solitary lake populations in North America, Norwegian freshwater sticklebacks had similar range in Tpos and α values, but much less trait divergences.

**Conclusions:**

Our results showed trait divergences between threespine stickleback in marine and freshwater environments. Freshwater populations diverged in trophic ecology and trophic traits, but trophic ecology was not related to the elapsed time in freshwater. Norwegian sticklebacks used the same niches as the ecologically divergent North American stickleback species pairs. However, as trophic trait divergences were smaller, and not strongly associated with the ecological niche, ecological adaptations along the benthic-limnetic axis were less developed in Norwegian sticklebacks.

## Background

Adaptive radiations in which there is a close association between niche use and trophic traits have intrigued scientists for centuries [1, 2]. Here, subtle to strong trophic specialization and habitat use associations are indicators of early to late adaptive steps in taxa such as European whitefish (*Coregonus lavaretus*) [3, 4] and threespine stickleback (*Gasterosteus aculeatus*) [5]. However, the time scale for adaptation in wild populations is usually not known. Further, not all taxa show adaptive radiations. Thus, it is important to study mechanisms leading to adaptive radiations, ideally where the time scale for natural selection is known or can be inferred.

In the northern hemisphere post-glacial isostatic rebound has created freshwater lakes of varying ages providing an upper bound on timing of colonization and how long immigrants could be adapting to the lake environment [6]. The ancestral marine threespine stickleback was one such taxon invading freshwater systems as they became available after the retreat of the Pleistocene ice-sheet, representing a remarkable example of parallel evolution and adaptive radiation into these new freshwater environments [7–9]. Many attempts have been made to understand the selective landscape leading to the diversification of freshwater sticklebacks [8–11]. Current adaptations in freshwater sticklebacks may stem from standing genetic variation in the marine population, from selective colonization of individuals preadapted to freshwater, or from new adaptive mutations and selective processes that have arisen post-glacially (0–20 000 years before present) [12, 13]. Few studies have specifically addressed the origin and rate of novel adaptive phenotypic diversity during this postglacial process. These studies show that phenotypic change may occur rapidly even on a contemporary time scale [14–16]. Strong selection on threespine stickleback phenotypes in new environments has been documented [17, 18]. Furthermore, evolutionary constraints due to allometric scaling relationships have been suggested to partly shape phenotypic diversification in postglacial Norwegian freshwater threespine sticklebacks [19], setting a limit to the extent and rate of phenotypic change.

Even though estimates on trait diversification and selection in threespine sticklebacks exists we still need information on trophic ecology associated with trait changes to understand if and how ecological conditions may affect the rate of adaptive diversification in freshwater lakes. Few comparative studies of trophic transitions between marine and freshwater environments are available for the threespine stickleback. However, the ancestral marine sticklebacks are assumed to be pelagic foragers in a homogeneous environment [8, 20], while anadromous sticklebacks could also spend a large proportion of the year in the ocean feeding on pelagic zooplankton [21]. Conversely, freshwater sticklebacks are assumed to live in a much more heterogeneous environment than populations in marine habitats and to feed on various prey sources in the littoral and pelagic area, e.g. [22]. Thus, divergent niche use between marine and freshwater stickleback, and among freshwater stickleback populations, may lead to selection for different adaptive trophic morphologies in these contrasting environments [1, 10, 22].

Most Holarctic stickleback populations usually contain trophic generalist individuals. In such solitary generalist populations (i.e. one trophic form of stickleback present), phenotypic differentiation along the benthic-limnetic axis (i.e. putative morphological adaptation from littoral-benthic to pelagic areas) can be observed both within and among populations [23, 24]. In a small subset of lakes, sticklebacks have diverged into benthic and limnetic morphs (referred to as species pairs) [1, 25]. Here, contrasting adaptations have accompanied occupation of benthic and limnetic niches with ecological selection against intermediate phenotypes [26, 27]. The limnetic morph forages mainly in the pelagic habitat, having a long fusiform body, large eyes, a long slender head, a terminal mouth and many long gillrakers [7, 21]. In contrast, the benthic morph uses a more spatially complex environment, feeds on larger invertebrates, and has a deeper body and head, a shorter and wider mouth, and fewer and shorter gillrakers [7, 27]. Interestingly, the phenotypic diversification in the solitary lake populations of the threespine stickleback seem to resemble trophic adaptations in the benthic-limnetic species pair [7, 27], with variable position along this axis in different lakes suggesting ongoing trophic adaptation. Matthews et al. [20] used stable isotopes to compare generalist and benthic-limnetic stickleback morphs/species pairs, and found that divergence in habitat use coincided with shifts in trophic position. The limnetic morph had a higher trophic position than the benthic morph. Sticklebacks with longer gillrakers obtained less carbon from benthic sources. Matthews et al. [20] implied that gillraker length was a key trait permitting adaptive niche shifts among lake habitats. Gillraker length is associated with food preference, prey items and stable isotopes in sticklebacks [28], as may be the case for gillraker numbers but to a lesser extent [29]. Thus, freshwater stickleback populations display a wide range of phenotypes ranging from generalists to specialized forms that exploit the benthic-limnetic niche axis, providing an excellent model for studies on rapid post-glacial trophic adaptations.

Individual freshwater systems differ considerably in terms of their morphology and abiotic and biotic conditions, and thus the shape and intensity of natural selection on trophic traits and foraging behavior in the threespine stickleback differ among systems. Individuals in generalist threespine stickleback populations with phenotypes more adapted to the benthic or limnetic niche seem to have higher fitness than those with intermediate morphology [30, 31]. In such, “optimal phenotypes” in a freshwater system may depend on lake morphometry and niche-specific availability of benthic and limnetic prey [30]. Here, intraspecific resource competition can be an important driver for foraging-related phenotypic divergence and niche variation [31]. This adaptive process will likely be affected by piscivorous predators - setting the frame for ecological opportunity [25, 30–32].

In this study, we used a set of twentyfive freshwater and one marine threespine stickleback populations to compare trophic phenotypes between environments in Norway. The main goal was to test specifically if, and to what degree, the freshwater sticklebacks showed trophic trait adaptations to lake niches in the timeframe following the Pleistocene deglaciation. This task was accomplished by first comparing adaptive trophic trait variation (head shape, gill raker number and gill raker length) between the marine and freshwater stickleback populations. Secondly, we tested if and how this trophic trait variation was associated with trophic ecology in freshwater lakes, particularly looking for association with the age of the postglacial lakes. Further, we tested if trophic ecology was associated with the age of postglacial lakes. More specifically, geometric morphometrics were used to characterise head shape, and we counted the number of gillrakers, and measured gillraker length. We used nitrogen stable isotope values (δ^15^N) of littoral and pelagic invertebrates to estimate individual and population trophic position (Tpos), and carbon stable isotope values (δ^13^C) to estimate the proportion of dietary carbon from littoral sources (α) as an estimate of long-term habitat use. Furthermore, lake parameters (estimated mean depth and distance to the sea), and estimates of freshwater lake age (when lakes were formed after deglaciation – due to specific isostatic uplift patterns) were used for testing for associations between morphology, habitat use (α) and trophic position (Tpos). We further compared the Norwegian threespine stickleback populations to two sets of the highly specialized benthic and limnetic species pair from North American lakes. Finally, we tested if the stickleback populations in these Norwegian lakes showed similar trait diversity as in the benthic-limnetic species pairs and illuminated how niche use was associated with morphology in Norwegian and a set of North American stickleback populations. Thus, our study approach was to test trophic trait divergence between marine and freshwater lakes, with an emphasis on how trophic ecology and trophic trait associations were potentially modulated by lake specific parameters in the available time frame for evolution in Norwegian postglacial lakes. The comparison between Norwegian sticklebacks and the North American benthic-limnetic species pairs was done to describe the extent of diversification, and the degree of similarity, in associations between trophic traits, niche use and trophic position in a continuum of generalist to species-pair stickleback lakes on these two continents.

## Methods

### Study area and sample collection

**Fig. 1:**
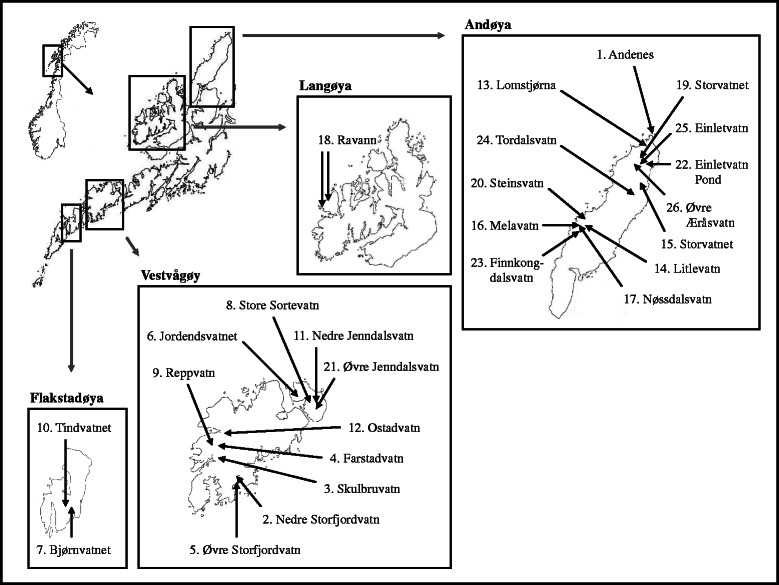
Sample collection information of the threespine stickleback localities. The marine (1. Andenes harbor) and freshwater (*n* = 25) Norwegian threespine stickleback populations studied from the area of Lofoten including the islands of Andøya, Langøya, Flakstadøya and Vestvågøy. See Table 2 for additional geographical- and lake information

During August 2006, threespine sticklebacks were sampled from 26 populations (one marine coastal sample, 25 freshwater lakes) from the Island of Andøya and from different islands in the Lofoten archipelago, NW Norway (Fig. 1, Table 1). Marine fish were collected using small mesh-sized gillnets in the Andenes harbour. Freshwater sticklebacks were collected using a standardized set of plexiglass traps set in the shallow (< 2 m depth) littoral. After sampling, fish were stored in 70 % EtOH after being euthanized using an overdose of MS222.

**Table 1:** Information on analysed Norwegian threespined sticklebacks populations. N denotes total (males/females). See Fig. 1 for map

Population	Island	Drainage	N (M/F)	Estimated age (ybp)	Elevation (m a. s. l.)	Area (km^2^)	Distance to sea (m)	Mean depth (m)	Fish species	Body length cm mean (std)
1. Andenes Harbor	Ocean	Ocean	20 (10/10)	0	0	-	-	-	Several taxa	5.32 (0.31)
2. Nedre Storfjordvatn	Vestvågøy	Storfjordelva	20 (4/16)	3 100	3	0.114	265	4.61	Bt	4.55 (0.35)
3. Skulbruvatn	Vestvågøy	Skulbruelva	19 (11/8)	3 300	3	0.141	290	5.02	As, Bt, Ac	4.45 (0.42)
4. Farstadvatn	Vestvågøy	Farstadelva	19 (7/12)	3 300	3	1.250	1197	12.03	As, Bt, Ac	4.37 (0.69)
5. Øvre Storfjordvatn	Vestvågøy	Storfjordelva	20 (2/18)	5 400	7	0.185	610	5.60	Bt	4.44 (0.45)
6. Jordendsvatnet	Gimsøy	Jordendselva	20 (5/15)	12 500	6	0.121	1579	4.73	Bt	4.52 (0.35)
7. Bjørnvatnet	Flakstadøya	Rishaugelva	19 (8/11)	12 500	26	0.023	1728	2.43	Bt	3.97 (0.21)
8. Store Sortevatn	Gimsøy	Sorteelva	20 (6/14)	13 500	13	0.217	1047	5.97	Bt	4.28 (0.32)
9. Reppvatn	Vestvågøy	Farstadelva	20 (2/18)	13 600	9	0.572	863	8.80	As, Bt, Ac	3.97 (0.54)
10. Tindvatnet	Flakstadøya	Nussfjordelva	19 (11/8)	13 800	44	0.052	2470	3.37	Bt	3.87 (0.40)
11. Nedre Jenndalsvatn	Gimsøy	Jenndalselva	19 (8/11)	14 300	20	0.216	1087	5.96	Bt	4.73 (0.35)
12. Ostadvatn	Vestvågøy	Farstadelva	19 (5/14)	14 600	23	1.223	7893	11.92	As, Bt, Ac	4.26 (0.60)
13. Lomstjørna	Andøya	Tofteelva	20 (6/14)	14 700	24	0.029	2595	2.67	As, Bt	4.49 (0.34)
14. Litlevatn	Andøya	Kryssdalselva	20 (10/10)	14 900	35	0.063	7086	3.64	Bt	4.67 (0.47)
15. Storvatnet	Andøya	Storeelva	20 (7/13)	15 000	26	0.310	2360	6.89	As, Bt, Ac	4.27 (0.45)
16. Melavatn	Andøya	Melaelva	20 (4/16)	15 300	13	1.968	3698	14.42	As, Bt, Ac	4.62 (0.51)
17. Nøssdalsvatn	Andøya	Melaelva	20 (7/13)	15 400	22	0.969	6788	10.86	As, Bt, Ac	4.24 (0.58)
18. Ravatn	Langøya	Nykvågelva	20 (2/18)	15 500	10	0.267	800	6.49	Bt, Ac	4.42 (0.39)
19. Storvatnet	Andøya	Rambergselva	20 (3/17)	15 500	30	1.000	3379	11.00	As, Bt	4.43 (0.53)
20. Steinsvatn	Andøya	Steinvasselva	20 (6/14)	15 500	16	1.599	3832	13.27	Bt	4.41 (0.36)
21. Øvre Jenndalsvatn	Gimsøy	Jenndalselva	19 (9/10)	15 500	40	0.268	2409	6.50	Bt, Ac	4.16 (0.25)
22. Einletvatn Pond	Andøya	Litleelva	20 (15/5)	16 000	37	0.007	3561	1.51	-	4.70 (0.50)
23. Finnkongdalsvatn	Andøya	Melaelva	20 (8/12)	16 500	41	0.139	8500	5.00	Bt	3.84 (0.58)
24. Tordalsvatn	Andøya	Nordelva	19 (5/14)	17 000	43	0.737	4648	9.74	Bt, Ac	3.82 (0.35)
25. Einletvatn	Andøya	Litleelva	15 (6/9)	18 000	37	0.282	3520	6.63	Bt	4.56 (0.38)
26. Øvre Æråsvatn	Andøya	Tofteelva	19 (6/13)	18 700	44	0.244	5300	6.26	Bt, Ac	4.63 (0.33)

Species codes in column “Fish species”; *As* Atlantic salmon (*Salmo salar*), *Bt* Brown trout (*Salmo trutta*), *Ac* Arctic char (*Salvelinus alpinus*)

For each lake we estimated lake surface area (A; km^2^) from maps and used this to estimate the mean depth based on an empirical model of data from 488 Norwegian lakes [33]. First, lake volume (V) was estimated as V = 0.011A^1.4^. Mean depth (D; m) was then estimated as D = 1000*V/A e.g. [34]. As area and depth parameters are not independent, we used only estimated mean depth for further statistical analyses. Information on the presence of fish predators was obtained from local fishers organizations. Both piscivorous fish and birds may predate on threespine stickleback, and in such impose selection pressures affecting niche use in sticklebacks, but information on birds predating on sticklebacks do not exist in these lakes. Thus, we have used piscivore fish as a general description of predation regime. The piscivore predators in this part of Norway are Atlantic salmon (*Salmo salar*), brown trout (*Salmo trutta*), arctic charr (*Salvelinus alpinus*) and European eel (*Anguilla anguilla*). Brown trout were present in all lakes except one (Einletvatn Pond) (Table 1).

All lakes in the region were formed following postglacial isostatic uplift. The age of each lake was estimated using the Sea level 3.51 software [6] (Table 1), and found to vary from 3 100 to 18 700 years before present (ybp). For five lakes situated above the last glacial marine limit, we used glacial isochrones based on the recession of the ice margin [35] to estimate the upper bounds for the putative timing of stickleback colonization of a lake. As we do not have information regarding exact timing of stickleback colonization, lake age sets an upper time frame for colonization. We assume that marine stickleback retain their ancestral morphological state and assign this population an age of zero. Marine sticklebacks tend to have relatively similar morphology while brackish and freshwater stickleback often differ [21, 36, 37].

In order to visualize the extent of phenotypic diversity in the Norwegian sticklebacks we compared our marine and freshwater populations to the most phenotypically and ecologically divergent sympatric benthic-limnetic species pairs known globally, using Paxton and Priest Lake, British Colombia, Canada [38]. Secondly, this approach visualized how far Norwegian stickleback populations had diverged along the phenotypic-ecological benthic-limnetic axis compared to the North American benthic-limnetic species pairs. Furthermore, another aim was to describe if divergence along this axis in Norwegian freshwater sticklebacks resembled the phenotypic niche divergence seen in the North American benthic-limnetic species pairs. Here, we used head shape as an axis of comparison (see detailed description below). The sticklebacks from Priest and Paxton lakes were collected using minnow traps set in the littoral zone during May-June 2005 [38]. Paxton Lake fish included 37 limnetic morph individuals (24 males and 13 females) and 27 benthic morph individuals (15 males and 12 females), whereas the dataset from Priest Lake comprised 24 limnetic morph individuals (24 males and 0 females) and 40 benthic morph individuals (27 males and 13 females).

### Trophic ecology and habitat use: stable isotope analysis

For the Norwegian freshwater sticklebacks we assessed diet and niche use with stable isotope analysis (SIA) of carbon (δ^13^C) and nitrogen (δ^15^N) ratios. Stickleback δ^13^C values provide information on use of prey along the limnetic-littoral (benthic) resource axis with ^13^C-depleted values reflecting utilization of limnetic prey and ^13^C-enriched values indicating consumption of littoral (benthic) prey. These isotopes are typically correlated with the long-term diet e.g. [39]. Furthermore, δ^15^N values provide information on the trophic level of stickleback [40]. No information is available for nitrogen isotope turnover in sticklebacks, but Grey [41] showed that δ^13^C data generally reflect the dietary/feeding history during the last 6 months. This has also been found for other temperate fishes [42].

From each Norwegian freshwater stickleback a piece of the tail muscle was extracted and dried for 24 h at 60 °C, ground, weighed, encapsulated in tin cups and analysed with a stable isotope ratio mass spectrometer following Harrod et al. [43]. Muscle C:N ratios differed among populations (ANOVA: F_25, 488_ = 61.3, *P* < 0.0001), indicating variation in lipid concentrations. Thus, the δ^13^C data were arithmetically lipid-normalised before further analysis [44]. To provide information on isotopic relationships of limnetic and benthic (littoral) resources from each lake, we sampled zooplankton in the limnetic zone using standard plankton nets and littoral (benthic) chironomids using standardized kick sampling with a hand-held dip net [45]. Zooplankton and chironomids were treated as two groups although the species composition within and between lakes may differ. The isotopic endpoints were estimated from the mean δ^13^C values from littoral/benthic and limnetic prey from each lake, and trophic fractionation in carbon (δ^13^C) was estimated at 1‰ [40]. We further calculated the trophic position (Tpos) and the proportion of carbon obtained from littoral sources (α) applying formulas 1 and 2 in Post [40] (also used in [20]).

The measures we used to compare the trophic position (Tpos) and habitat use (α) of sticklebacks from Norway and North America include corrections for site-specific differences in isotopic baselines e.g. of littoral and open water habitats, allowing direct comparison of results from the two continents. Stable isotopic data (population means and standard deviations) for North American stickleback populations were found in Matthews et al. [20].

### Trophic traits

Norwegian marine and freshwater sticklebacks were sexed by internal examination of gonads. Total fish length was measured to the nearest mm, the number of gillrakers on the first right gill arch was counted, and the length of the third gillraker on the lower gill arch was measured using a stereo microscope. These traits, together with head shape, are important for food acquisition in sticklebacks [46–48], and often correlate with stomach content [28, 31], stable isotope values [28], and foraging efficiency on divergent prey items see [49–51].

**Fig. 2:**
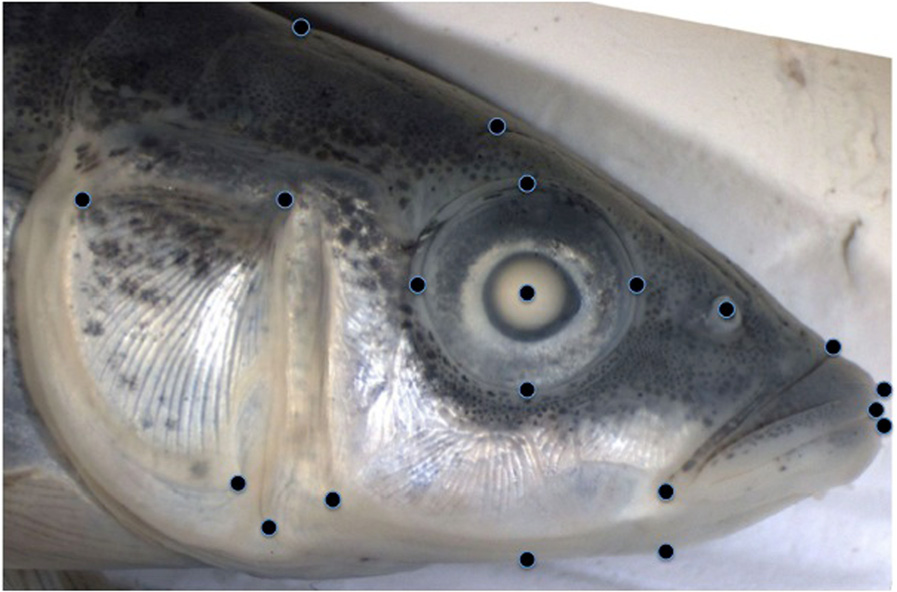
Distribution of the 20 landmarks used for head shape analyses in the threespine stickleback (displayed on a representative marine stickleback from Andenes harbour, Andøya, Norway)

A digital image was captured of the lateral side of the head of Norwegian stickleback individuals when immersed in 70 % EtOH using a CANON EOS 350D digital camera with a 90 mm lens (Tamron macro). D. Schluter provided a comparative dataset of pictures for North American sticklebacks. The Norwegian and North American pictures have been gathered by two different persons (K. Østbye and by courtesy of R. Svanbäck). In order to quantify and characterize the variation in head shape among sticklebacks we first applied a geometric morphometric analysis of the pictures using the thin plate spline (Tps) morphometric software TspDig2 [52]. Here, 20 landmarks (Fig. 2) were selected to describe the integrated shape of the head and digitized. All the landmarks were set by one person (K. Østbye). The landmark data were subsequently imported into the software MorphoJ 1.01b [53, 54] where we performed a Procrustes fit and secondly performed a principal component analysis (PCA) saving the most important principal components for our analyses (see below).

### Statistical analysis

First, we tested if a set of trophic traits (head shape - based on the extracted principal components, gillraker number and gillraker length) in Norwegian threespine sticklebacks differed between the marine and freshwater habitats using a general linear mixed model (glmm) with habitat (marine/freshwater), body length and sex as fixed effects and lake as a random factor. This analysis was conducted to visualise potential trophic trait differences between the assumed ancestral marine and the derived freshwater populations.

Secondly, we quantified trophic trait diversification among all the Norwegian freshwater threespine stickleback populations. Head shape was analysed using principal components and the most important PC-axes were extracted. Then, we used glmm to examine how variation in principal component traits, as well as variation in gillraker number and length, was predicted by individual variation in two related habitat use proxies (the proportion of somatic carbon derived from littoral sources (α) and trophic position (Tpos)) and fish characteristics (body length (cm) and sex). Lake identity was used as a random factor in the analysis.

Further, we used glmm to test how trophic position (Tpos) and the proportion of carbon from littoral sources (α) varied among Norwegian freshwater populations, and how these measures were related to lake age, mean depth of lakes, and distance to the sea, using sex and body length as additional predictors. Lake identity was used as a random factor in the analysis.

To visualise diversity in head shape we compared the Norwegian marine and freshwater populations with the benthic-limnetic species pairs in North America using a joint principal component analysis. This analysis produced two informative principal components, where PC1 differentiated the Norwegian and North American stickleback while PC2 differentiated the divergent species pairs in North America (see details in the result section). Thus, we used the polarization along PC2 (hereafter called trophic axis) to evaluate trait differences of the Norwegian stickleback along the “benthic-limnetic phenotypic (associated with niche) axis”.

By using the trophic axis estimates for the Norwegian freshwater sticklebacks we used glmm to test if this axis was correlated with gillraker length and number, mean lake depth, proportion of organic carbon from littoral sources (α), trophic position (Tpos), sex and length (cm), with lake identity as a random factor. The idea was to test if similar trait changes as seen in the benthic-limnetic North American species pairs could be found in solitary Norwegian stickleback populations.

All statistical analyses were conducted using the software package JMP 9.0 [55].

## Results

### Freshwater lake characteristics

Most of the Norwegian lakes investigated here were small (mean ± sd: 0.48 ± 0.54 km^2^), shallow (mean depth: 7.0 ± 3.6 m), and situated at low elevation (23.0 ± 14.2 m above sea level). The distance to the sea ranged between 0.27 and 8.5 km (3.1 ± 2.4 km). However, the lakes covered the whole zone below the post-glacial upper - maximum marine level (Table 1). Most lakes were relatively old for the region (13 372 ± 4 536 years) with a minimum age of 3 100 years and a maximum age of 18 700 years: four lakes were formed less than 10 000 years ago.

### Trophic trait differences between marine and freshwater stickleback

**Fig. 3:**
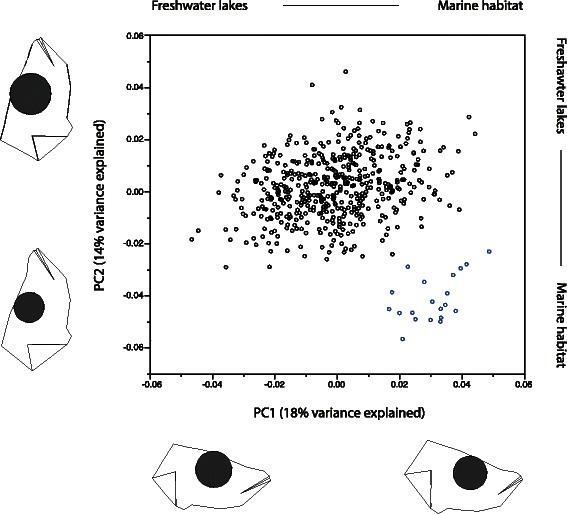
Comparison of head shape among Norwegian marine and freshwater threespine sticklebacks. Landmark-based head shape is given for PC1 versus PC2. The interpreted head shape (based on analysis in software MorphoJ) represents fish at the extreme of the respective distributions on both axes. Blue symbols indicate marine fish from the island of Andøya (Andenes harbour) while black symbols represents freshwater lake populations

In the principal component analysis of the Norwegian marine and freshwater populations we used the first four components explaining 18.4 % (PC1, Eigenvalue = 0.00028), 13.8 % (PC2, 0.00021), 11.7 % (PC3, 0.00018), and 8.6 % (PC4, 0.00013), of the shape variation, respectively. There were significant differences between marine and freshwater stickleback for PC1, PC2 and PC3 (Table 2, Fig. 3). Based on these three PC-axes, the marine sticklebacks had a more upturned mouth, smaller eye lower on the head, larger operculum, and deeper head than freshwater sticklebacks. Gillraker number did not differ between environments. However, gillrakers were significantly longer in marine than freshwater sticklebacks and males had slightly more and longer gillrakers than females (Table 2).

**Table 2:** Summary results from general linear mixed models testing for differences in head morphology (overall head shape extracted from a principal component analysis, gillraker number and gillraker length) between threespine sticklebacks from marine and the freshwater habitats

Test	Source	DF	Estimate ± SE	*F*	*P*
Head shape (PC1)	Intercept		0.019 ± 0.008		0.021
R^2^ = 0.62	Habitat (marine vs freshwater)	1	−0.017 ± 0.006	8.07	0.009
N fish = 504	Sex	1	0.004 ± 0.000	55.22	< 0.001
N populations = 26	Total length (cm)	1	−0.001 ± 0.001	0.89	0.345
Head shape (PC2)	Intercept		0.024 ± 0.005		< 0.001
R^2^ = 0.63	Habitat (marine vs freshwater)	1	0.016 ± 0.003	38.06	< 0.001
N fish = 504	Sex	1	0.005 ± 0.000	126.20	< 0.001
N populations = 26	Total length (cm)	1	−0.009 ± 0.001	95.62	< 0.001
Head shape (PC3)	Intercept		−0.014 ± 0.006		0.027
R^2^ = 0.25	Habitat (marine vs freshwater)	1	−0.006 ± 0.002	4.78	0.038
N fish = 504	Sex	1	0.003 ± 0.001	28.92	< 0.001
N populations = 26	Total length (cm)	1	0.004 ± 0.001	12.30	0.005
Head shape (PC4)	Intercept		−0.027 ± 0.006		< 0.001
R^2^ = 0.37	Habitat (marine vs freshwater)	1	0.005 ± 0.003	2.24	0.147
N fish = 504	Sex	1	−0.001 ± 0.001	1.47	0.227
N populations = 26	Total length (cm)	1	0.006 ± 0.001	26.92	< 0.001
Gillraker number	Intercept		18.897 ± 0.683		< 0.001
R^2^ = 0.29	Habitat (marine vs freshwater)	1	−0.078 ± 0.374	0.04	0.836
N fish = 503	Sex	1	−0.185 ± 0.058	10.17	0.002
N populations = 26	Total length (cm)	1	0.048 ± 0.119	0.16	0.686
Gillraker length	Intercept		0.380 ± 0.060		< 0.001
R^2^ = 0.61	Habitat	1	−0.177 ± 0.028	35.85	< 0.001
N fish = 503	Sex	1	−0.043 ± 0.005	62.71	< 0.001
N populations = 26	Total length (cm)	1	0.137 ± 0.011	155.21	< 0.001

Total length (cm) and sex used as covariates. Lake identity was used as a random variable

### Habitat use and trophic position related to lake characteristics

**Fig. 4:**
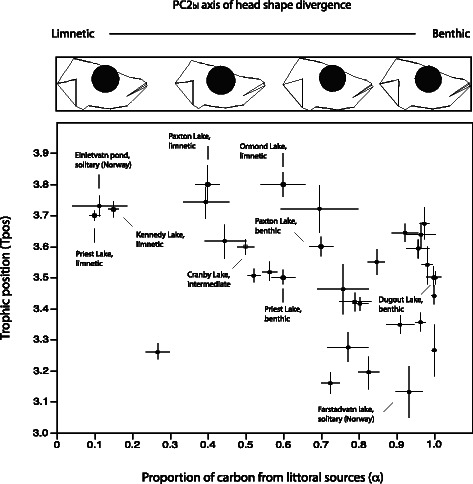
Comparison of trophic level and littoral carbon in the diet in North American and Norwegian threespine sticklebacks. Mean (± SE) trophic position (Tpos) versus the proportion of littoral carbon in the diet (α) in twentyfive Norwegian freshwater stickleback populations (males and females pooled). The eight North American stickleback populations from Matthews et al. [20] are included for comparison of generalist to specialist populations. Here we have used the same ecological categorization of populations as in Matthews et al. [20], spanning limnetic, benthic and intermediate ecophenotypes. Two of the Norwegian populations are named for visual comparison of range in Tpos and α, namely the Einletvatn pond (limnetic ecophenotype) and Farstadvatn lake (benthic ecophenotype). In the top section, from left to right, are given head shape along the benthic-limnetic trophic axis (PC2 in Fig. 5) for Lake Einletvatn Pond, Lake Paxton-limnetic, Lake Paxton-benthic and Lake Farstadvatn. The depicted head shapes represents mean values from each of the populations

Stable isotope analysis revealed that Norwegian freshwater sticklebacks obtained on average 76 % (range 11–100 %) of their carbon (α) from littoral sources, having a mean trophic level (Tpos) of 3.47 ± 0.27 (range 3.13–3.74), with large variation among the freshwater stickleback populations (Fig. 4). Only Tpos was significantly and positively associated with total length, and males had a significantly higher trophic position (Tpos) (least square mean (LSM = 3.50) than did females (LSM = 3.45) (Table 3). However, the proportion of littoral carbon (α) in the diet was not related to any of the predictor variables.

**Table 3:** Summary results from general linear mixed models testing for differences in the proportion of organic carbon obtained from littoral sources (α) and estimated trophic position (Tpos) of threespine sticklebacks from 25 freshwater locations

Test	Source	DF	Estimate ± SE	*F*	*P*
Littoral carbon (α)	Intercept		0.981 ± 0.207		< 0.001
R^2^ = 0.69	Sex	1	0.002 ± 0.009	0.06	0.814
N fish = 482	Total length (cm)	1	−0.030 ± 0.018	2.69	0.102
N populations = 25	Lake age (years)	1	−0.000 ± 0.000	1.06	0.314
	Distance to sea (m)	1	−0.000 ± 0.000	1.57	0.224
	Mean depth (m)	1	−0.000 ± 0.015	0.00	0.992
Trophic position (Tpos)	Intercept		2.762 ± 0.165		< 0.001
R^2^ = 0.51	Sex	1	−0.028 ± 0.010	7.82	0.005
N fish = 482	Total length (cm)	1	0.135 ± 0.021	42.53	< 0.001
N populations = 25	Lake age (years)	1	0.000 ± 0.000	2.80	0.109
	Distance to sea (m)	1	−0.000 ± 0.000	0.11	0.740
	Mean depth (m)	1	−0.010 ± 0.011	0.94	0.343

The habitat characteristics lake age (years before present), mean depth (m), and distance to the sea (m) and individual total length (cm) and sex were predictors. Lake identity was used as a random variable

### Trophic traits, habitat use and trophic position in freshwater

In the principal component analysis contrasting head shape among the Norwegian freshwater stickleback lakes the first four principal components explained 19.1 % (PC1, Eigenvalue = 0.00027), 13.0 % (PC2, 0.00019), 9.6 % (PC3, 0.00014), and 8.1 % (PC4, 0.00011) of the variation. Neither of the principal components were significantly related to any of the two habitat use indices (Tpos, α), but there was a significant effect of sex (Table 4). Gillraker number and length differed between males and females, where males had more and longer gillrakers than females. Gillraker length also increased with an increasing proportion of carbon (α) from littoral sources (Table 4).

**Table 4:** Summary results from general linear mixed models testing for differences in head morphology (extracted from the principal component analysis, gillraker number and gillraker length) of threespine sticklebacks from 25 freshwater locations

Test	Source	DF	Estimate ± SE	*F*	*P*
Head shape (PC1)	Intercept		0.023 ± 0.011		0.038
R^2^ = 0.57	Littoral carbon (α)	1	−0.004 ± 0.003	1.76	0.185
N fish = 482	Trophic position (Tpos)	1	−0.000 ± 0.003	0.04	0.833
N populations = 25	Sex	1	0.005 ± 0.001	79.20	< 0.001
	Total length (cm)	1	−0.004 ± 0.001	12.42	< 0.001
Head shape (PC2)	Intercept		−0.011 ± 0.011		0.295
R^2^ = 0.25	Littoral carbon (α)	1	0.004 ± 0.003	1.64	0.203
N fish = 482	Trophic position (Tpos)	1	0.002 ± 0.003	0.71	0.401
N populations = 25	Sex	1	0.005 ± 0.000	58.81	< 0.001
	Total length (cm)	1	−0.000 ± 0.001	0.04	0.836
Head shape (PC3)	Intercept		−0.039 ± 0.009		< 0.001
R^2^ = 0.42	Littoral carbon (α)	1	−0.002 ± 0.002	0.99	0.322
N fish = 482	Trophic position (Tpos)	1	0.002 ± 0.002	0.53	0.467
N populations = 25	Sex	1	−0.000 ± 0.000	3.64	0.057
	Total length (cm)	1	0.008 ± 0.001	65.20	< 0.001
Head shape (PC4)	Intercept		0.018 ± 0.008		0.036
R^2^ = 0.33	Littoral carbon (α)	1	−0.000 ± 0.002	0.00	0.988
N fish = 482	Trophic position (Tpos)	1	0.002 ± 0.002	0.61	0.435
N populations = 25	Sex	1	0.000 ± 0.000	1.63	0.203
	Total length (cm)	1	−0.005 ± 0.001	30.25	< 0.001
Gillraker number	Intercept		19.474 ± 1.055		< 0.001
R^2^ = 0.30	Littoral carbon (α)	1	−0.176 ± 0.294	0.36	0.550
N fish = 481	Trophic position (Tpos)	1	−0.153 ± 0.274	0.31	0.578
N populations = 25	Sex	1	−0.173 ± 0.060	8.34	0.004
	Total length (cm)	1	0.050 ± 0.125	0.16	0.693
Gillraker length	Intercept		0.190 ± 0.095		0.046
R^2^ = 0.42	Littoral carbon (α)	1	0.075 ± 0.026	8.41	0.004
N fish = 481	Trophic position (Tpos)	1	−0.016 ± 0.025	0.42	0.515
N populations = 25	Sex	1	−0.045 ± 0.006	67.84	< 0.001
	Total length (cm)	1	0.139 ± 0.011	148.80	< 0.001

Habitat use (proportion of organic carbon extracted from littoral sources (α), trophic position (Tpos)), total length (cm) and sex were used as factors. Lake identity was used as a random variable

### Head shape in marine ancestor, generalists and species pairs

**Fig. 5:**
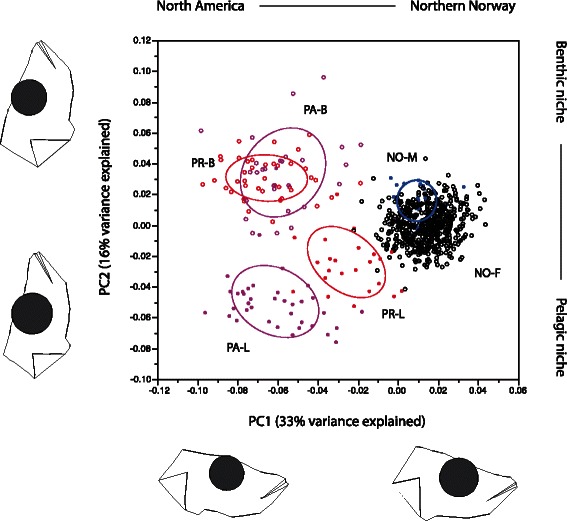
Comparison of head shape in North American and Norwegian threespine sticklebacks. Landmark-based head shape (PC1 versus PC2) in one Norwegian marine (blue symbols) and twentyfive freshwater stickleback populations (black symbols) compared with to sets of the North American ecologically divergent species pairs (Lake Paxton (pink symbols) and Priest lake (red symbols)). Circles denotes 50 % ellipses of each group. Population-morphs codes are as follows: Priest lake benthic (PR-B), Priest lake limnetic (PR-L), Paxton lake benthic (PA-B), Paxton lake limnetic (PA-L), Norwegian marine population at the island of Andøya (NO-M) and the 25 freshwater lakes (NO-F). The head shape (based on MorphoJ analyses) represents individuals at outer margins of the distribution on both axis

In the joint principal component analysis contrasting Norwegian marine and freshwater sticklebacks with North American species pairs, the first two components explained 33.2 % (PC1, Eigenvalue = 0.00096), and 16.3 % (PC2, 0.00047) of the shape variation, respectively. Here, PC1 differentiated the sticklebacks from the two continents, while PC2 mainly differentiated the ecologically divergent species-pairs along the benthic-limnetic shape axis (Fig. 5). Thus, we used PC2 as morphological polarization (hereafter termed “trophic axis”) to test for associated changes in Norwegian freshwater sticklebacks along the same axis as in North American species pairs.

In Norwegian freshwater sticklebacks, head shape on the trophic axis was significantly associated with sex, where males were more limnetic in head shape than females. The trophic axis was also significantly, and positively, associated with body length, but negatively associated with gillraker length and gillraker number (Table 5). Moreover, the mean lake depth was also negatively associated with the trophic axis. Thus, individuals with a “limnetic morph” head shape had more gillrakers as well as longer gillrakers than individuals with a more “benthic morph” head shape. The “limnetic morph” was thus associated with deeper lakes.

**Table 5:** Summary results from a general linear mixed model for threespine sticklebacks from 25 freshwater locations testing for associations along the trophic axis (i.e. head shape axis in PC2 in Fig. 5) with predictor factors gillraker length and number, mean depth of lake, proportion of organic carbon obtained from littoral sources (α), trophic position (Tpos), sex and length (cm)

Test	Source	DF	Estimate ± SE	*F*	*P*
Trophic axis	Intercept		0.004 ± 0.012		0.727
R^2^ = 0.41	Gillraker length (mm)	1	−0.019 ± 0.004	18.44	< 0.001
N fish = 481	Gillraker number	1	−0.001 ± 0.000	6.43	0.012
N populations = 25	Mean depth (m)	1	−0.001 ± 0.000	6.55	0.018
	α	1	0.000 ± 0.003	0.00	0.967
	Tpos	1	0.001 ± 0.003	0.26	0.612
	Sex	1	0.002 ± 0.001	10.65	0.001
	Total length (cm)	1	0.007 ± 0.001	32.57	< 0.001

Lake identity was used as a random variable

## Discussion

### Trait transitions between the sea and freshwater environments

In our study, head shape in Norwegian freshwater populations differed significantly from the marine population. In general, marine fish had a more upturned mouth, a smaller eye lower on the head, a larger operculum, and a deeper head when compared with freshwater sticklebacks. The divergent head shape in our marine and freshwater sticklebacks was qualitatively similarly to that found by Voje et al. [19] who studied 11 marine and 74 freshwater stickleback populations in Norway. This head shape divergence between the two contrasting environments could imply functionally different trophic adaptations to divergent niches, although we do not have comparative stable isotopic data to support our claim. Previous studies comparing marine and freshwater sticklebacks are thus only partly consistent with our data showing a divergent (but contrasting head shape) of marine and freshwater populations in both North America [38, 56, 57] and Europe [37, 58]. We are not aware of studies that have analysed both trophic traits and niche use in marine and freshwater sticklebacks. This issue should be studied to reach a needed contrast of trophic ecology and trophic trait associations.

In our study, gillraker counts were similar in marine and freshwater habitats. This finding is supported by Kristjánsson et al. [59] who compared marine and freshwater populations of sticklebacks on Iceland and by Berner et al. [10] comparing marine and freshwater sticklebacks in North America. Overall, only moderate differentiation has been reported for gillraker number in populations from contrasting habitat types [60, 61]. Apparently, the adaptive transition from marine to freshwater habitats may not necessarily involve changes in gillraker number.

The Norwegian marine population had significantly longer gillrakers than freshwater sticklebacks, supporting similar observations from North America [10] and Europe [59, 61]. This divergence may reflect the more pelagic lifestyle of the marine stickleback [8]. Moreover, Gross & Anderson [61] observed that inter-raker spacing was smaller in marine populations than in freshwater populations, suggesting a pattern where marine sticklebacks have more and longer rakers and thus a denser gillraker apparatus. Only the length of gill rakers differed in our marine-freshwater comparison. Svanbäck & Schluter [38] found that solitary generalist populations showed more variation in gillraker length than did either marine ancestors or the benthic-limnetic species pair. The genetic basis for such phenotypic divergence has been found by common garden experiments [22, 38, 62–64], also revealing plasticity for gillraker length [38]. Berner et al. [10] found that divergence in phenotypic (co)variance structure in marine and freshwater stickleback populations was large and attributable to shifts in variance of gillraker lengths. Thus, gillraker length seems to underlie adaptive changes in divergent environments [20], and may be free to evolve without strong ties from the ancestral (co)variance structure upon freshwater colonization, but see also [22].

We have found that Norwegian marine and freshwater stickleback differ significantly in trophic traits (head shape and gill raker length). The degree and rate of phenotypic diversification in these environments could be due to a number of factors. A study by Voje et al. [19] compared a larger set of Norwegian populations using a model of niche-dependent optima to study trait changes along the spatio-temporal axis in postglacial sticklebacks. In that study, the results suggested that populations diversified in phenotypic directions due to allometric scaling relationships, further implying that evolutionary constraints had shaped phenotypic diversification of freshwater populations. Our Norwegian threespine stickleback populations were sampled from the same regions as in Voje et al. [19], which may suggest that the same interpretations could partly be valid also for our dataset.

### Habitat use and trophic position - lake variables and trophic morphology

The freshwater sticklebacks studied here obtained most of their carbon from littoral sources, suggesting that they largely used benthic and littoral niches. However, at least one population was highly divergent from this pattern, being largely dependent on carbon from the pelagic niche, implying that pelagic foraging specialists occurs in some of these Norwegian lakes. This particular population (Einletvatn pond) had a higher trophic position and smaller α than the other Norwegian populations, suggesting a more pelagic lifestyle. The head shape of this population resembles the head shape of the limnetic morph members of the North American species pairs (Fig. 4), but with a lower degree of shape differentiation.

The associations between habitat use and trophic position in the Norwegian freshwater stickleback was complex. First, we found that habitat use (α) was associated with gillraker length where sticklebacks with longer gillrakers had a larger contribution of carbon from littoral sources than sticklebacks with shorter gillrakers. This is opposite to what was predicted by Matthews et al. [20]. This finding could partly be due to differential niche-adaptation loads with regard to fitness reduction in an alternative niche by having a specific trophic trait adapted to a certain niche. In such, sticklebacks with longer gill rakers could forage effectively also on larger benthic prey sources, whereas sticklebacks with smaller gill rakers would be adapted to the benthic niche and relatively less efficient in the limnetic niche. In such, niche-specific trophic trait adaptations may have different fitness costs in alternative environments such as in the benthic and limnetic niche. Secondly, if we assume that the ancestral marine trophic state is to forage on pelagic resources, and that freshwater lakes have less profitable pelagic niches, we should expect that older stickleback populations in freshwater lakes should prefer to forage in the benthic niche (higher α). However, this seems not to be the case as α was not associated with lake age. This finding imply that Norwegian freshwater sticklebacks, despite their long time in postglacial lakes, behaves as generalists (using both benthic and limnetic niches) and have not had the ecological opportunity or time to specialize to either the benthic or limnetic niche. Here, rewarding food resources in the limnetic niche may only be temporally available during yearly variable summer months – a resource which would be difficult to adapt to. Further, we found an association between trophic position and size; larger sticklebacks had a higher trophic level than smaller sticklebacks, implying a broader niche with age. Moreover, males were foraging at a higher trophic level than females, a finding supporting earlier results [65].

### Changes along the benthic-limnetic trophic axis of divergence

To compare Norwegian freshwater and marine stickleback populations in their morphological adaptation along the commonly discussed benthic-limnetic axis, we compared our data with the ecologically highly specialized benthic-limnetic species pairs in Paxton and Priest lakes. Here, we tested if similar trait associations as seen for these diverged species-pairs were also present in sticklebacks in Norwegian lakes. We found that the Norwegian freshwater populations in general had very low variation along this trophic axis, being intermediate to the North American species pairs (Fig. 5; PC2). However, some Norwegian populations tended to be more “limnetic” and some were more “benthic” compared with the North American species-pairs (Fig. 4; head shape). A similar quantitative differentiation among freshwater lakes in head shape was found by Willaker et al. [24] in North American stickleback populations. Interestingly, head shape in Norwegian lakes was negatively associated with mean lake depth – fish from deeper lakes had a more “limnetic” head, which could be due to a larger relative pelagic zone in these lakes as compared to the smaller and shallower lakes. In support of this, Willaker et al. [24] found that more shallow lakes had more “benthic” shaped sticklebacks in North American lakes. In shallow lakes, it is likely that benthic production overwhelms limnetic production [66–68] and thus regulates the availability of divergent niches.

We found, similarly as in the North American species-pairs, more numerous and longer gill rakers in fish with a limnetic head shape. In our study, body size was significantly and positively associated with head shape, further suggesting that larger fish with more benthic head shapes had a more benthic lifestyle. However, neither α nor Tpos was associated with head shape, which implies that the apparent adaptive transitions between the benthic and limnetic niche (as suggested by head shape in species-pairs) do not need to result in differences in trophic trait changes in individuals. This strategy could be due to temporal use of the limnetic zone in Norwegian sticklebacks. Piscivorous predators were present in all but one (Einletvatn Pond) of our Norwegian stickleback lakes, and this could constrain habitat use of the stickelback. In Willaker et al. [24], the presence of salmonids did not have a significant impact on the shape of sticklebacks, but lakes with pike (*Esox lucius*) had more “limnetic” shaped sticklebacks. Thus, it is seems likely that different piscivore fish species exerts differential selection pressures in different lake habitats upon threespine stickleback phenotypes.

### Evolutionary origin of stickleback morphs in post-glacial lakes

The Norwegian freshwater stickleback utilize carbon sources spanning the whole range from almost 0 % to 100 % littoral origin. When comparing this with the North American solitary populations and species pairs (including Paxton and Priest Lakes) published by Matthews et al. [20], we see that the solitary Norwegian populations span a wide range of niche use and trophic position compared with the North American populations (see Fig 4). The Norwegian sticklebacks appear overall to be more benthic (mean α = 0.76 ± 0.29, *N* = 486) than North American species-pairs (0.45 ± 0.31, *N* = 403) (Fig. 4). Norwegian sticklebacks seem to have a slightly lower trophic position (mean Tpos; 3.47 ± 0.27, *N* =4 86) than North American sticklebacks (3.60 ± 0.23, *N* = 403). The wide range in Tpos between Norwegian stickleback populations implies that the populations utilize as diverse ecological niches as the highly diverged benthic-limnetic species pairs in North America. The Norwegian population with the highest trophic level and the smallest α value was a very small pond population (Einletvatn pond) where stickleback was the only fish species present. This population resembles limnetic North American forms (Fig. 4; head shape). These results suggest that the Norwegian sticklebacks have utilized ecologically all the foraging niches typically available to sticklebacks in a given lake (i.e. benthic and limnetic niches), but with only low correlation between trophic trait specialization and their utilized diet sources (Tpos and α).

Of the numerous freshwater lakes studied throughout the Holarctic only a handful of lakes harbour specialized species pairs that have radiated to the full extent into the littoral and pelagic niches, while the major number of stickleback lakes are populated by solitary generalists [1, 7, 8, 19, 21]. Why have so few stickleback populations diversified into these niches following postglacial colonization? A set of hypotheses can be suggested. First, it may be that the occurrence of the benthic-limnetic species pairs in North America, being confined to a geographically restricted area in the straits of Georgia in the Vancouver area, is due to a special set of glacial geological events that have not occurred frequently elsewhere. This hypothesis, supported by genetic studies [69], suggests that two waves of marine ancestors colonized the lakes at different time periods. In our Norwegian lakes, we do not have data to evaluate these possibilities. Another hypothesis under the double-colonization-scenario is that the ecological opportunity to radiate into the littoral and pelagic habitat is physically possible, but that an extensive ecological radiation is constrained by some factors. One possibility here would be that the piscivorous predators restrict sticklebacks from utilizing the pelagic zone. We have no data at hand to evaluate this hypothesis.

Lakes harbouring benthic-limnetic species pairs have a very specific fish fauna compared to lakes with single generalist populations [25]. Vamosi [25] found that solitary populations and sympatric benthic-limnetic species pairs had different fish fauna. In lakes with benthic-limnetic species pairs only cutthroat trout (*Oncorhynchus clarki*) were present, while in 15 of 16 solitary lakes other fish species in addition to cutthroat trout were found. The two most common species in solitary lakes were prickly sculpin (*Cottus asper*) and rainbow trout (*Oncorhynchus mykiss*), both potential competitors and predators of threespine sticklebacks. In our Norwegian lakes, piscivorous fish such as brown trout and arctic charr occur frequently, where only one location is missing other fish species (Einletvatn Pond). This pond population showed the highest ecological similarity with the limnetic species pair members in North America (Fig. 4). The occurrence of predators could restrict the postglacial adaptive radiation of sticklebacks.

Another scenario that may explain stickleback diversity in Holarctic lakes is adaptive divergence after colonization of a single stickleback wave at the time of glaciostatic uplift. Here, it is reasonable to expect a longer evolutionary time needed to differentiate the original population into divergent littoral and limnetic niches. In this situation, a driving force of adaptive split into niches may be intraspecific resource competition. The degree of resource competition may be a function of the stickleback population density. However, this scenario is related to the theoretical challenges for initial within-population divergence and assortative mating to develop [1, 2, 70]. This may be a reason for the lack of a large diversity of stickleback morphs, or species, in the Holarctic, because divergence of populations needs assortative mating linked with niche divergence to develop and retain reproductive barriers.

An important issue with regard to stickleback diversity in Holarctic lakes relates to the genetic potential for adaptive tracking of available niches upon postglacial colonization. A study has suggested presence of evolutionary constraints in colonizing sticklebacks where phenotypic diversification largely followed the allometric scaling relationships for the studied traits when comparing both marine and freshwater lake populations in Norway [19]. Our stickleback populations are sampled within the same region as in Voje [19]. North American and Norwegian stickleback populations could harbour different genetic architectures and allometric scaling relationships and be differentially predisposed to radiate into the benthic and limnetic niche in freshwater lakes. However, as analyses such as done by Voje et al. [19] are not done on the North American sticklebacks we have no means of contrasting the adaptive potential.

To our knowledge, no benthic-limnetic species pair of the threespine stickleback has been found in Europe. However, the small number of sympatric benthic-limnetic stickleback pairs in a small geographical area in North America points to a special geographical occurrence in that particular area leading to the formation of the species pairs (i.e. double-immigration-scenario). If this is the case, we can imagine that the evolution of the North American species pairs was facilitated by the separation time (and adaptation) of the two species pairs before coming into secondary contact in the same lake. In such, expecting that solitary populations in the Holarctic, that likely have evolved by a one-colonization-scenario, should exhibit similar adaptive differentiation along the benthic-limnetic niche continuum as seen in the species pairs seems unrealistic. This is the situation since the North American species pair may have been formed by a different evolutionary scenario initially. Indeed, the few occurrences of benthic-limnetic species pairs as compared to the hundreds of Holarctic stickleback lakes thus imply that the limited extent of adaptive trophic trait evolution along the benthic-limnetic axis is a general phenomena for most of the Holarctic stickleback lakes. Thus, the solitary generalist populations and the species-pairs may represent specializations along the adaptation-speciation continuum, but with different evolutionary initial conditions.

## Conclusions

Norwegian sticklebacks utilized the full spectrum of foraging niches, and seem to have the same range in trophic position as North American solitary populations and species pairs. However, Norwegian sticklebacks showed less developed and associated trait adaptations, but, if contrasted along the trophic axis, similar trait changes were seen among continents. The Norwegian sticklebacks can be viewed as solitary generalist where some populations display foraging-related niche specialization. The position along the adaptive trophic axis in Norwegian solitary populations and North American benthic-limnetic species pairs are likely due to two initially different evolutionary scenarios. Here, benthic-limnetic species pairs are likely formed due to a double-immigration-scenario, while most solitary Holarctic stickleback populations likely represent one-immigration-scenarios. The studied Norwegian stickleback lakes were up to 18 000 years old (6 000–18 000 stickleback generations assuming a 3–1 year life cycle). However, lake age showed no association with ecological divergence (Tpos, α). This postglacial timeframe of putative evolution is similar to the North American stickleback lakes. The mechanisms behind the origin of stickleback morphs with different initial starting conditions (i.e. double and one colonization scenarios) should be contrasted in future studies.

### Ethical approval

Permit for sampling of stickleback individuals were given by the Norwegian Directorate for nature management (permit no 10/06). Other permits are not required.

### Consent to publish

Not required.

### Availability of data and materials

The dataset) supporting the conclusions of this article are available in the Dryad Digital Repository: http://dx.doi.org/10.5061/dryad.qp512.
